# Editorial: Intelligent analysis of biomedical imaging data for precision medicine

**DOI:** 10.3389/fmed.2022.1017751

**Published:** 2022-09-26

**Authors:** Kuanquan Wang, Shuo Li, Xiuying Wang, Jun Feng, Yong Xu

**Affiliations:** ^1^Faculty of Computing, Harbin Institute of Technology, Harbin, China; ^2^Department of Biomedical Engineering, Case Western Reserve University, Cleveland, OH, United States; ^3^Faculty of Engineering, School of Computer Science, The University of Sydney, Sydney, NSW, Australia; ^4^School of Information Science and Technology, Northwest University, Xi'an, China

**Keywords:** biomedical imaging, intelligent analysis, classification, segmentation (image processing), deep learning—artificial neural network

Biomedical imaging, such as X-ray, ultrasound, computed tomography (CT), magnetic resonance (MR), positron emission tomography (PET), and microscopic imaging, has been widely applied in clinical practices due to its capabilities in depicting physical anatomy and revealing functional and biochemical process of the human body. Biomedical images are becoming indispensable for more accurate diagnosis and precision medicine including pre-/intraoperative planning, survival prediction, and evaluation of the therapeutic response. However, the accuracy of the diagnostic decisions often heavily relies on the experience of radiologists, and thereafter inevitably is subject to inter-/intra-operator variations. Besides, due to the limitations of imaging techniques, the image artifacts, inhomogeneity in intensity, and low contrast between tissues also impede the accurate diagnosis. Thus, there is a demand to design intelligent analysis methods of biomedical imaging data for more efficient, objective, and effective precision medicine.

Intelligent analysis methods, based on biomedical imaging data, are designed to provide quantitative and qualitative evaluation for auxiliary diagnosis. In this Research Topic, the intelligent analysis methods are used to segment and recognize the region of interest (ROI) in different organs and tissues (Zhang T. et al., Peng et al., Ma et al., Li D. et al., Bo et al., Chen H. et al., Zhang D. et al., Zhang J. et al., He et al., Wang et al.), evaluate the histological risk (Han et al., Li D. et al., Bo et al., Yang Y. et al., Xiao et al.), locate, count and classify cells (Li H. et al., Song et al.), reconstruct and visualize the three-dimensional model (Cao et al.), predict the progression-free survival (Chen N. et al.) and measure organs (Yang C. et al.).

For evaluation of histological risk types, Han et al. introduced the acute angle between adjacent lobulations as a new quantitative indicator for the prediction of the risk classification on thymomas. In their method, the least absolute shrinkage and selection operator (LASSO) was adopted to make the feature selection and the individualized imaging nomogram was used to evaluate the prediction ability of the selected feature. The authors verified that the acute angle was significantly associated with the risk classification (*p* < 0.05). For improving the diagnostic ability of prostate cancer, Li D. et al. divided the deep learning model into three parts: prostate gland segmentation, classification, and prostate cancer area segmentation. First of all, the prostate gland segmentation network was implemented to acquire the mask of the gland, based on which, the mask was moreover cropped on ADC, DWI, and T2WI sequences. Secondly, the prostate classification network was executed to determine whether the cropped area contained prostate cancer. Once the gland was abnormal, the prostate cancer area segmentation network was finally applied to segment the lesion area. The experimental results showed that all the accuracy, precision, and sensitivity had obtained an evident improvement. Bo et al. combined deep transfer learning with hand-crafted radionics features for classification between brain abscess and cystic glioma, which achieved higher accuracy than deep learning or hand-crafted based model. To understand the relationship between the chronic obstructive pulmonary disease (COPD) risks and age, Yang Y. et al. separated the subjects by COPD stages. Within each group, the age data is divided into eight equal intervals. Then, the survival Cox model was created on lung radionics features to estimate the risk probability of COPD. The evaluation metrics area under curve showed the excellent performance of the proposed model. To enrich the image and provide more details for the clinical diagnosis of lung cancer, Xiao et al. proposed a siamese pyramid fusion network for PET images and CT images fusion (PET-CT) to simultaneously display the metabolic and anatomical information. In order to validate the improvement of image fusion, the authors used five classification methods (multlayer perceptron, support vector classifier, random forest, K-nearest neighbor, and naive Bayes classifier) for training and testing on PET, CT, and PET-CT. The experimental data demonstrated that all the models based on PET-CT data obtained an evident improvement compared to PET and CT data.

For brain tumor segmentation, Zhang T. et al. creatively applied the wormhole theory on quantum-behaved particle swarm optimization (QPSO) for dealing with smeared and irregular shapes in medical image segmentation tasks. Even with the low contrast and high inhomogeneity in the medical image, the proposed QWPSO method recovered the contours of the tumor well which were consistent with the ground truth. In recent years, coronavirus disease 19 (COVID-19) has been spread around the world. Peng et al. designed an ensemble model consisting of deep supervised learning networks (DeepLab V3+, U-Net, PAN, and FPN) for COVID-19 lesion segmentation. The proposed ensemble model achieved a better segmentation result than manual segmentation as 0.7279 in IOU metric and 92.4604 in Hausdorff distance metric. As the lymph nodes are highly relative to lung adenocarcinoma, it is necessary to identify lymph nodes from CT to make a better diagnosis and treatment. To identify the lymph node accurately, Ma et al. constructed a cost-sensitive uncertainty hypergraph learning (CSUHL) scheme. On the one hand, both epistemic and aleatoric uncertainty were adopted to improve the quality of pathological representation. A new hypergraph-based learning scheme was used to reconsider the correlation between samples to generate high-order representations. On the other hand, the scheme was devised to capture the cost sensitivity of negative samples and assign more weights to the lymph node. The loss function let the model focus on the patient with lung adenocarcinoma. In the experiment phase, the proposed model obtained the highest prediction accuracy in 0.95238 among state-of-the-art methods. It is evidently better than the method BC-GNN which has the second highest prediction accuracy in 0.91667.

Intelligent medical image segmentation has also become important in the orthopedic area. As the manual segmentation for knee bone and cartilage is tedious and subjective, Chen H. et al. chose a three-dimensional deep neural network (nnU-Net) as their baseline model, and the adversarial loss was selected to provide the prior shape constraints and expand the contextual receptive filed for resampled volume segmentation. The proposed method was assessed on the public dataset SKI10 and achieved a score of more than 76 in the validation phase. It was proven that the method can either extract the area of healthy bone and cartilage accurately or the pathological cases. Similar to research in Chen H. et al., He et al. introduced an adversarial learning scheme into 3D U-Net. Additionally, a Squeeze and Excitation (SE) module was added to increase the weight of relevant features and decrease the weight of irrelevant features for liver segmentation. As for the malignant melanoma recognition tasks in a whole-slide image (WSI) with huge size, Zhang D. et al. first broke up the WSI into several patches to relieve the computational burdens. Then, the location information, predicted categories, as well as confident probabilities, were combined to acquire the recognition result of malignant melanoma. Thirdly, as the pathological features appear on different scales, a multi-scale feature fusion architecture was designed to enrich the lesion features. Meanwhile, for irregularly shaped lesion areas, the deformable convolution style residual blocks and channel attention mechanism were constructed to focus on the essential features and reduce the influence of noise.

For intelligent medical image analysis methods, segmenting the thin structure from low contrast and ambiguous images is still a great challenge. Zhang J. et al. attempted to fuse multi-scale features within each layer called intra-layer pyramid-scale aggregation blocks (IPABs). The blocks generated two associations at both high and low scales. Besides, the pyramid skip connection and deep pyramid supervision were used for further enhancement. The performance of the proposed block was verified in three public datasets (DRIVE, STARE, and CHASE-DB1). The experimental data showed that the proposed block can effectively extract thin vessels and outperformed the current state-of-the-art methods. As biomedical images often come from different domains, directly applying the same intelligent analysis model to different domains may cause poor prediction results. To relieve this problem, Wang et al. put forward to model of the semi-supervised learning approach and unsupervised domain adaptation approach into the same framework. And generative adversarial network changed to predict the label for each pixel for leveraging the annotated and unannotated data in the segmentation task here.

Intelligent diagnosis of a pathological image often demands counting the number of positive cells to estimate the illness state. Li H. et al. selected the two-stage feature pyramid network as the baseline model. The anchor-based model predicted the categories and refined the anchors several times which is more suitable for cell counting tasks. Some researchers created an intelligent analysis method for predicting progress-free survival (PFS) in patients with cancer. For example, Chen N. et al. explored radiomics signatures from the contrast-enhanced CT images to predict the PFS of a patient with small cell lung cancer. At first, the image features, including shape, intensity, texture, and so on, were calculated from each tumor area. The univariate prognostic ability of features was estimated by Cox proportional hazard (CPH) model. Then, the variation inflation factor (VIF) method was performed to remove the redundant features. Random survival forests (RSFs) were further applied to simplify the features. Only the features with a high importance score could be selected to form the radiomics signature. Based on selected radiomics and clinical features, the prognostic model was created. Finally, 11 radiomics features are selected and the model can predict the PFS with high accuracy.

After reviewing this Research Topic, we conclude that the intelligent analysis of biomedical image data for precision medicine can improve the diagnosis procedure in a more efficient and scientific way. The featured researchers are devoted to obtaining more accurate diagnosis results for specialized clinical problems, and the experiment results show that their method is positive examples of precision medicine, which indicates that the methods could be assisted in making personalized clinical plans for diagnosis in the future.

## Author contributions

KW wrote the overall framework of the paper and summarized the contents of the edited articles. SL, XW, JF, and YX summarized the contents of their respective edited articles. All authors contributed to the article and approved the submitted version.

## Funding

This work was supported by the National Natural Science Foundation of China under Grant 62272135.

## Conflict of interest

The authors declare that the research was conducted in the absence of any commercial or financial relationships that could be construed as a potential conflict of interest.

## Publisher's note

All claims expressed in this article are solely those of the authors and do not necessarily represent those of their affiliated organizations, or those of the publisher, the editors and the reviewers. Any product that may be evaluated in this article, or claim that may be made by its manufacturer, is not guaranteed or endorsed by the publisher.

